# Supplementary material for: A UMLS-based spell checker for natural language processing in vaccine safety
Source: BMC Med Inform Decis Mak. 2007 Feb 12;7:3. doi: 10.1186/1472-6947-7-3 (PMC1805499; doi:10.1186/1472-6947-7-3)
Supplement: Additional file 13 — RAP application directory. Sets up directory for RAP (RDF application for PHP) source code [file 1472-6947-7-3-S13.gz › rap/doc/phpdoc/__filesource/fsource_default__vocabularyRDFS_C.php.html]

xml version="1.0" encoding="iso-8859-1"?


File Source for RDFS\_C.php


# Source for file RDFS\_C.php

Documentation is available at RDFS\_C.php

```
1. <?PHP

3. // ----------------------------------------------------------------------------------
4. // RDF Vocabulary Description Language 1.0: RDF Schema (RDFS) Vocabulary (Resource)
5. // ----------------------------------------------------------------------------------
6. // Version                   : 0.9
7. // Authors                   : Daniel Westphal (dawe@gmx.de)
8. //
9. // Description               : Wrapper, defining resources for all terms of the
10. //                               RDF Schema (RDFS).
11. //                               For details about RDF see: http://www.w3.org/TR/rdf-schema/.
12. //                                Using the wrapper allows you to define all aspects of
13. //                             the vocabulary in one spot, simplifing implementation and
14. //                             maintainence.
15. //
16. // ----------------------------------------------------------------------------------
17. // History:
18. // 02-21-2003                 : Initial version
19. // ----------------------------------------------------------------------------------

21. // RDFS concepts

23. class RDFS{

25. function RESOURCE()
26. {
27. return  new Resource(RDF_SCHEMA_URI . 'Resource');

29. }

31. function LITERAL()
32. {
33. return  new Resource(RDF_SCHEMA_URI . 'Literal');

35. }

37. function RDFS_CLASS()
38. {
39. return  new Resource(RDF_SCHEMA_URI . 'Class');

41. }

43. function DATATYPE()
44. {
45. return  new Resource(RDF_SCHEMA_URI . 'Datatype');

47. }

49. function CONTAINER()
50. {
51. return  new Resource(RDF_SCHEMA_URI . 'Container');

53. }

55. function CONTAINER_MEMBERSHIP_PROPERTY()
56. {
57. return  new Resource(RDF_SCHEMA_URI . 'ContainerMembershipProperty');

59. }

61. function SUB_CLASS_OF()
62. {
63. return  new Resource(RDF_SCHEMA_URI . 'subClassOf');

65. }

67. function SUB_PROPERTY_OF()
68. {
69. return  new Resource(RDF_SCHEMA_URI . 'subPropertyOf');

71. }

73. function DOMAIN()
74. {
75. return  new Resource(RDF_SCHEMA_URI . 'domain');

77. }

79. function RANGE()
80. {
81. return  new Resource(RDF_SCHEMA_URI . 'range');

83. }

85. function LABEL()
86. {
87. return  new Resource(RDF_SCHEMA_URI . 'label');

89. }

91. function COMMENT()
92. {
93. return  new Resource(RDF_SCHEMA_URI . 'comment');

95. }

97. function MEMBER()
98. {
99. return  new Resource(RDF_SCHEMA_URI . 'member');

101. }

103. function SEEALSO()
104. {
105. return  new Resource(RDF_SCHEMA_URI . 'seeAlso');

107. }

109. function IS_DEFINED_BY()
110. {
111. return  new Resource(RDF_SCHEMA_URI . 'isDefinedBy');
112. }

114. }
115. ?>
```

Documentation generated on Fri, 17 Dec 2004 16:17:16 +0100 by phpDocumentor 1.3.0RC3
